# A Sonic Hedgehog-Gli-Bmi1 signaling pathway plays a critical role in p27 deficiency induced bone anabolism

**DOI:** 10.7150/ijbs.65954

**Published:** 2022-01-01

**Authors:** Jun Wu, Rong Wang, Xuechun Kan, Jinghan Zhang, Wen Sun, David Goltzman, Dengshun Miao

**Affiliations:** 1 State Key Laboratory of Reproductive Medicine; Research Center for Bone and Stem Cells; Department of Anatomy, Histology and Embryology; Key Laboratory for Aging & Disease; Nanjing Medical University, Nanjing 211166, China.; 2 Calcium Research Laboratory, McGill University Health Centre and Department of Medicine, McGill University, Montreal, Quebec H3A 1A1, Canada.; 3 The Research Center for Aging, Affiliated Friendship Plastic Surgery Hospital of Nanjing Medical University, Nanjing 210029, China.

**Keywords:** p27, osteogenesis, osteoblast formation, sonic hedgehog signaling, Bmi1

## Abstract

To explore the mechanism of the bone anabolic action of p27 deficiency, we first confirmed that osteoblast formation and osteogenesis were significantly increased in p27 deficient mice compared with their wild-type littermates. Microarray analysis of differential gene expression profiles, followed by real-time RT-PCR and Western blots revealed that p27 deletion significantly upregulated the expression of Sonic hedgehog (Shh), Gli1 and 2 and their target gene Bmi1 in bone tissue, and significantly down regulated the expression of the negative regulators of the Shh pathway Sufu, Patched 1 and Gli3 in bone tissue. The Shh antagonist KAAD-cyclopamine or vismodegib significantly reduced osteogenesis of bone marrow mesenchymal stem cells (BM-MSCs) *in vitro* and osteoblastic bone formation *in vivo*. The results of chromatin immunoprecipitation and double luciferase assay demonstrated that p27 inhibited Shh transcription mediated via E2F4. Bmi1 knockout blocked the increase of osteoblastic bone formation induced by p27 deficiency *in vivo*. In conclusion, the results of this study indicate that the signaling pathway Shh-Gli-Bmi1 plays a critical role in p27 deficiency induced bone anabolic action, suggesting that Bmi1 may be an important therapeutic target for osteoporosis induced by activation of p27 signaling or inactivation of sonic hedgehog signaling.

## Introduction

The protein p27 (cyclin-dependent kinase inhibitor 1B or p27^Kip1^) is a member of the Cip/Kip family that also includes p21^Cip1^ and p57^Kip2^. Its classical role is to bind and inhibit multiple cyclin-dependent kinases involved in cell cycle progression 1. The importance of p27 as a cell cycle regulator *in vivo* was revealed by the main phenotypes of p27^-/-^ mice, namely increased body size, organ hyperplasia, pituitary tumors, and retinal dysplasia 2. In addition to these phenotypes, some studies have implied that p27 is likely a critical regulator of osteoblastic bone formation and osteogenesis. Bone marrow cells from p27^-/-^ mice exhibited increased proliferative activity and form an increased number and larger size of osteoblastic colonies, which can further differentiate to the stage of mineralization 3. Our previous studies demonstrated that p27 deletion significantly increased osteoblastic bone formation parameters in both long bones and mandibles, and improved the defective osteoblastic bone formation in 2-week-old mice lacking the nuclear localization sequence and C-terminus of PTH-related protein 4, 5. However, the detailed mechanism underlying p27 gene action in modulating osteoblastic bone formation is unclear.

Bmi1 (B-lymphoma Mo-MLV insertion region 1) is a key protein partner in Polycomb Repressive Complex 1 (PRC1) and acts as a transcriptional repressor of the Ink4a/Arf locus 6. Bmi1 knockout (Bmi1^-/-^) mice, established by homologous gene recombination technology, displayed a premature aging phenotype, which was associated with decreased self-renewal ability of neural stem cells and hematopoietic stem cells 7-9. Our studies demonstrated that Bmi1^-/-^ mice exhibited premature osteoporosis associated with reduced mesenchymal stem cell (MSC) self-renewal and decreased ability to differentiate into osteoblasts 10. We recently reported that overexpression of Bmi1 in MSCs exerts antiaging and antiosteoporosis effects by inactivating p16/p19 signaling and inhibiting oxidative stress 11. Bmi1 expression levels were up-regulated in bone tissue from p27 deficient mice 4, 5. However, it is unclear whether Bmi1 mediates p27 deficiency-induced bone anabolic action and how p27 regulates Bmi1 expression.

Overexpression of Bmi1 promotes cell proliferation and is required for pathway driven tumorigenesis. Bmi1 expression positively correlates with increasing Shh ligand concentrations. Chromatin immunoprecipitation reveals that Gli1 (glioma-associated oncogene1) preferentially binds to the Bmi1 promoter, and Bmi1 transcript levels are increased and decreased by Gli1 overexpression and downregulation, respectively 12. There is a potential nexus of Shh-Gli1-Bmi1 cell signaling to promote chemoresistance in glioma 13. The critical function of Shh signaling in bone formation has been identified in the past two decades. In the early stages of embryonic limb development, Shh acts as a major morphogen in patterning the limb buds 14. Shh is involved in intramembranous and endochondral ossification during fracture healing 15. Shh enhances osteogenesis of bone marrow MSCs (BM-MSCs) *in vitro* and *in vivo* 16-18. Previous studies have shown that haplo- or total insufficiency of p27 can induce the expression of Shh and increase the incidence of medulloblastoma 19; the down-regulation of the p27 gene in parathyroid carcinoma was always accompanied by upregulated Shh and Gli 20. Emerging evidence indicates that p27 also behaves as a transcriptional regulator. It associates with a number of gene promoters through E2F4/p130 complexes acting as a transcriptional repressor of these genes 2, 21-23. Therefore, we are investigated whether p27 might be recruited to the Shh promoter region to form p27/E2F4/p130 complexes, contributing to the repression of Shh signaling in osteogenic cells, and whether p27 deficiency stimulates osteoblast formation and osteogenesis by activating Shh-Gli-Bmi1 signaling.

To answer these questions, long bone phenotypes of 4-week-old p27 deficient and wild-type mice were compared to determine whether deletion of p27 stimulates osteoblast formation and osteogenesis. Microarray analyses of differential gene expression profiles were performed in long bone extracts from p27^-/-^ mice and their wild-type littermates to identify whether deletion of p27 activates Shh-Gli-Bmi1 signaling. The effects of an Shh antagonist or an Shh agonist on osteogenesis in p27 deficient or overexpressed BM-MSCs *in vitro*, and the effect of the Shh antagonist on osteoblastic bone formation in p27 deficient mice *in vivo* were determined to establish whether Shh signaling mediates p27 deficiency-induced osteogenesis and osteoblastic bone formation. We then generated p27^-/-^Bmi1^-/-^ double knockout mice, and their bone phenotypes were compared with wild-type littermates to determine whether deletion of Bmi1 blocks p27 deficiency-induced osteoblastic bone formation by blocking the Shh signaling pathway.

## Methods

### Mice and genotyping

Two mutant mouse models were used in this study: p27 heterozygous (p27^+/-^) mice were purchased from the Jackson Laboratory (Bar Harbor, ME, USA). Bmi1 heterozygous (Bmi1^+/-^) mice were generously provided by Dr. Anton Berns, of the Netherlands Cancer Institute. The p27 ^+/-^ mice and Bmi1^+/-^ mice were fertile and were mated to produce offspring heterozygous at both loci, which were then mated to generate p27^-/-^ Bmi1^-/-^ pups. All animal experiments were carried out in compliance with, and approval by, the Institutional Animal Care and Use Committee. The genotype of p27^-/-^ and Bmi1^-/-^ mice was confirmed as described previously 4, 24.

### Microarray analysis and real-time RT-PCR

RNA was isolated from mouse bone tissue, excluding the growth plate, using Trizol reagent (Invitrogen, Cat#15596026, Carlsbad, CA, USA) according to the manufacturer's protocol. RNA quality was assessed by Nanodrop ND-1000 and RNA integrity was assessed using standard denaturing agarose gel electrophoresis. The Mouse 12x135K gene expression array was manufactured by Roche NimbleGen. The microarray analysis was performed by KangChen Bio-tech. Total RNA was extracted from femurs or BM-MSCs using Trizol reagent (Invitrogen, Cat#15596026, Carlsbad, CA, USA) according to the manufacturer's protocol. Quantitative real-time RT-PCR amplifications were performed as previously described 5. Reverse-transcription reactions were performed using the PrimeScript RT Master Mix (Perfect Real Time, Takara Bio Inc., Cat#RR036D, Shiga, Japan). Real-time RT-PCR primers are listed in Table 1.

### Bone marrow mesenchymal stem cell (BM-MSC) cultures and cytochemical staining

Tibiae and femurs of wild-type and p27^-/-^ mice were removed under aseptic conditions, and bone marrow cells were flushed out with DMEM containing 10% FCS, 50 µg/mL ascorbic acid, 10 mM β-glycerophosphate, and 10^-8^ M dexamethasone. Cells were dispersed by repeated pipetting, and a single-cell suspension was achieved by forcefully expelling the cells through a 22-gauge syringe needle. Total bone marrow cells were cultured in six-well-plates at 10^6^ cells/well in 2 mL of the above-mentioned medium in the absence or presence of Shh-N (200 ng/ml) or Shh antagonist KAAD-Cyclopamine (KAAD,2.5μM). Medium was changed every 3 days, and cultures were maintained for 10 to 14 days. At the end of the culture period, cells were washed with PBS, fixed with PLP fixative, and stained cytochemically for ALP as described previously 25 to determine ALP-positive CFU-f (CFU-fap). After petri dishes were dried and imaged, they were stained with methyl blue to determine total CFU-f. Cellular senescence staining was performed with a cellular senescence β-galactosidase staining kit (# C0602, Beyotime Biotechnology, Wuhan, China) according to the manufacturer's protocol.

### Overexpression of p27 in BM-MSCs

BM-MSCs derived from WT mice were transfected with mouse p27-pCDNA3.1 (OV-p27) or vector plasmids which were purchased from Hanbio Co., Ltd. (Shanghai, China). BM-MSCs in the logarithmic phase were seeded into the 6-well plates (2 × 10^5^ cells/well) and cultured for 24 h. Cell transfection was then performed using Lipofectamine 3000® (Life Technologies, USA), cells were plated in 24-well plates and cultured with DMEM supplemented with 10% platelet lysate and 1% penicillin/streptomycin, and maintained under normal tissue culture conditions (37 °C, 5% CO_2_ in a humidified atmosphere) for 24 h before transfection. Two different Lipofectamine 3000® reagent and quantities of plasmid/DNA ratio were compared, and five cell confluences were tested. With the two best cell concentrations, four cell passages were compared. The protocol was performed according to the supplier's instructions. Briefly, plasmid DNA was mixed with OPTI-MEM (Life Technologies, USA) and the P3000 reagent (Life Biotechnologies, USA) in a microcentrifuge tube. In a separate tube, Lipofectamine 3000® reagent was diluted in OPTI-MEM. The contents of the two tubes were then combined by gentle pipetting and incubated at room temperature for 5 min, allowing the formation of DNA/lipid complexes. Then, the transfection mixture was added to the cells in culture. All transfection experiments were performed in triplicate.

### Iconographic and histopathological analyses

Femurs or tibias were removed and analyzed by radiography, micro-computed tomography, histology, histochemistry, and immunohistochemistry as we described previously 5, 26. Histochemical staining for ALP 27 and tartrate resistant acid phosphatase (TRAP) 28 were performed on the decalcified paraffin-embedded sections as we previously described. Immunohistochemistry was performed with the primary antibodies against Shh (ab135240; Abcam, Cambridge, MA, USA), Bmi1 (10832-1-AP; Proteintech, USA), and Gli2 (18989-1-AP; Proteintech, USA), Col-I (34710; Abcam, Cambridge, MA, USA).

### Immunocytochemistry

WT, Bmi1^-/-^ and p27^-/-^ BM-MSCs were seeded in 3.5 cm petri dishes and incubated at 37 °C with 5% humidification for 24 h. WT BM-MSCs were transfected with mouse p27-pCDNA3.1 (OV-p27) or vector plasmids. Then the BM-MSCs were treated with Shh-N (200 ng/ml) or with Shh antagonist KAAD-Cyclopamine (KAAD, 2.5 μM) for 10 days. After 10 days, culture medium was discarded. Immunocytochemistry was then performed as described previously 29 with the primary antibodies against Ki67 (ab15580; Abcam, Cambridge, MA, USA).

### Double calcein labeling

Double calcein labeling was performed by intraperitoneal injection of mice with 10 g calcein/g body weight (C-0875Sigma‐Aldrich, St. Louis, MO, USA) at 10 days and 3 days before death as described 30.

### Western blots

Proteins were extracted from bone tissue, and immunoblotting was carried out as we described previously 31. Immunoblotting was performed with the primary antibodies against Shh (ab135240; Abcam, Cambridge, MA, USA), Bmi1 (10832-1-AP; Proteintech, USA), Gli1 (ab49314; Abcam, Cambridge, MA, USA), Gli2 (18989-1-AP; Proteintech, USA), Gli3 (ab69838; Abcam, Cambridge, MA, USA), Ihh (ab39634; Abcam, Cambridge, MA, USA), Ptch1 (ab53715; Abcam, Cambridge, MA, USA), Sufu (#C54G2; Cell Signaling Technology, Beverly, MA, USA) and Smo (ab236465,Abcam, Cambridge, MA, USA). The β‐actin (BS6007M; Bioworld Technology, Bloomington, MN, USA) was used as the control for total protein.

### Chromatin immunoprecipitation

MEFs were plated in 20-cm plates and the cells grown to about 70% confluence. Chromatin immunoprecipitation was performed with the Magna ChIP^TM^ Chromatin Immunoprecipitation A kit (Millipore, Billerica, MA, USA, 2931149) according to the manufacturer's instruction. Chromatin samples were incubated with antibody against E2F4 (Millipore, MABE160, Billerica, MA, USA). The immunoprecipitated DNA samples were then analyzed by PCR and the samples were run on agarose (2%) gel electrophoresis for visualization. The primers used for the analysis of E2F4 binding are as follows:

- forward primer, 5-GGTACCGTGGCCACCTGTGATTATCC-3;
- reverse primer, 5- GAGCTCTGAGGACTTGTGAGCTGTCC-3.

### Dual luciferase assay

Mouse E2F4 gene was cloned into the vector pCDNA3.1 (Genechem Co., Ltd., Shanghai, China). For transfection experiments, the chimeric genes of the Shh promoter plasmid were constructed in a GV148-basic vector (Genechem Co., Ltd., Shanghai, China) by ligating the 5′-flanking regions of the mouse Shh gene upstream of the luciferase gene. Mouse MEF cells were plated into 24-well cell culture plates 1 day before transfection. The transfections included 1 µg each of pCDNA3.1-basic and GV148-basic; E2F4 over-expressing pCDNA3.1 and GV148-basic; E2F4 over-expressing pCDNA3.1 and GV148-mutant; E2F4 over-expressing pCDNA3.1 and GV148-Shh promoter; E2F4 over-expressing pCDNA3.1 and GV148-mutant, E2F4 over-expressing pCDNA3.1 and GV148-Shh promoter. These were respectively co-transfected with Firefly luciferase (Fluc) Renilla luciferase (Rluc) into mouse MEF cells using X-treme GENE HP DNA Transfection Reagent (Cat#6366236001, Roche Diagnostics Corp., Switzerland) according to the manufacturer's protocol. Two days later, the promoter-driven luciferase activity was measured using the Dual-Luciferase® Reporter Assay system (Cat# E1910, Promega Corporation, Madison, WI, USA).

### Statistical analysis

All analyses were performed using GraphPad Prism software (Version 6.07; GraphPad Software Inc., San Diego, CA, USA) as previously described (36). All statistical results are expressed as mean ± s.e.m. and are representative of at least three separate experiments (n > 6/group). Differences between two groups were analyzed using 2-tailed unpaired Student's *t*-test. P values < 0.05 were considered statistically significant.

## Results

### Deletion of p27 stimulates osteoblast formation and osteogenesis

**Figure 1:**
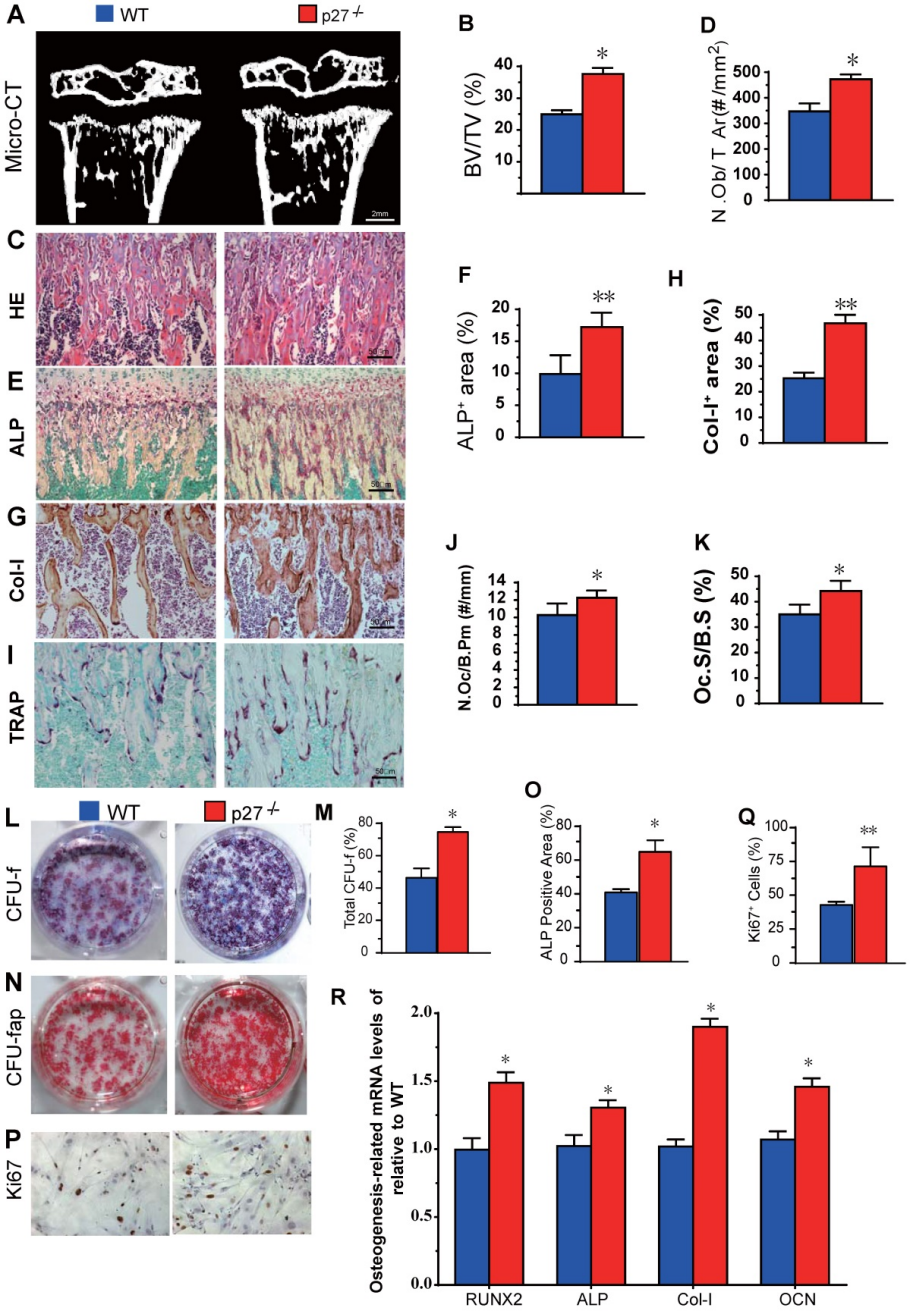
** Effect of p27 deficiency on osteoblastic bone formation and osteogenesis. (A)** Representative micro-CT scans of 3-dimensional longitudinal reconstructions of proximal ends of tibiae and midshaft diaphysis from 4-week-old WT and p27^-/-^ mice, and **(B)** Trabecular bone volume relative to tissue volume (BV/TV, %). Representative micrographs of paraffin-embedded sections of tibias from 4-week-old WT and p27^-/-^ mice showing **(C)** H&E staining and **(D)** the number of osteoblasts per mm^2^ tissue area (N.Ob/T.Ar, #/mm^2^), **(E)** histochemical staining for ALP and **(F)** ALP-positive areas as a percent of the tissue area (%), **(G)** immunostaining for type I collagen (Col-I), and **(H)** the percentages of Col-I positive areas, **(I)** histochemical staining for TRAP, **(J)** the number of osteoclasts/bone perimeter (Oc.N/B.Pm, #/mm) and **(K)** osteoclast surface/bone surface (Oc.S/BS, %). BM-MSCs from 4-week-old WT and p27^-/-^ mice were cultured *ex vivo* in osteogenic differentiation medium for 10 days. Resulting cultures were analyzed as follows: **(L)** staining with methyl blue for the total CFU-f and **(M)** total CFU-f-positive areas, **(N)** cytochemical staining for ALP to show CFU-fap and **(O)** ALP-positive areas, **(P)** immunocytochemical staining for Ki67 and **(Q)** the percentage of Ki67 positive cells. Real-time RT-PCR analyses of BM-MSC extracts for the expression of **(R)** *Runx2*, *ALP*, *Col-I* and *OCN*. Messenger RNA expression assessed by RT-PCR is expressed as a ratio relative to GAPDH expression. Values are mean ± s. e. m. of 5 determinations per group. *: *P*< 0.05; **: *P*< 0.01, compared with WT mice.

We first compared long bone phenotypes of 4-week-old p27 deficient and wild-type mice. The results revealed that bone volume (Figs. 1A & B), osteoblast numbers (Figs. 1C & D), alkaline phosphatase (ALP) positive area (Figs.1E & F) and type I collagen positive area (Figs. 1G & H) were all significantly increased, and osteoclast number and surface (Figs. 1I-K) were also increased in p27 deficient mice compared with wild-type littermates *in vivo*. We next examined BM-MSC cultures *ex vivo*. We found that total CFU-f, ALP positive CFU-f and the percentage of Ki67 positive cells were significantly increased (Figs. 1L-Q) and expression levels of the osteogenic genes, including *Runx2*, *ALP*, *type I collagen* and *osteocalcin* (*OCN*) were upregulated significantly (Figs. 1R) in BM-MSC cultures from p27 deficient mice compared with wild-type littermates. These results indicated that deletion of p27 could stimulate the proliferation of BM-MSCs and accelerate their osteoblastic differentiation, subsequently, increasing bone formation although deletion could also slightly increase osteoclasts.

### The Sonic hedgehog signaling pathway is activated in the bone tissue of p27 deficient mice

**Figure 2:**
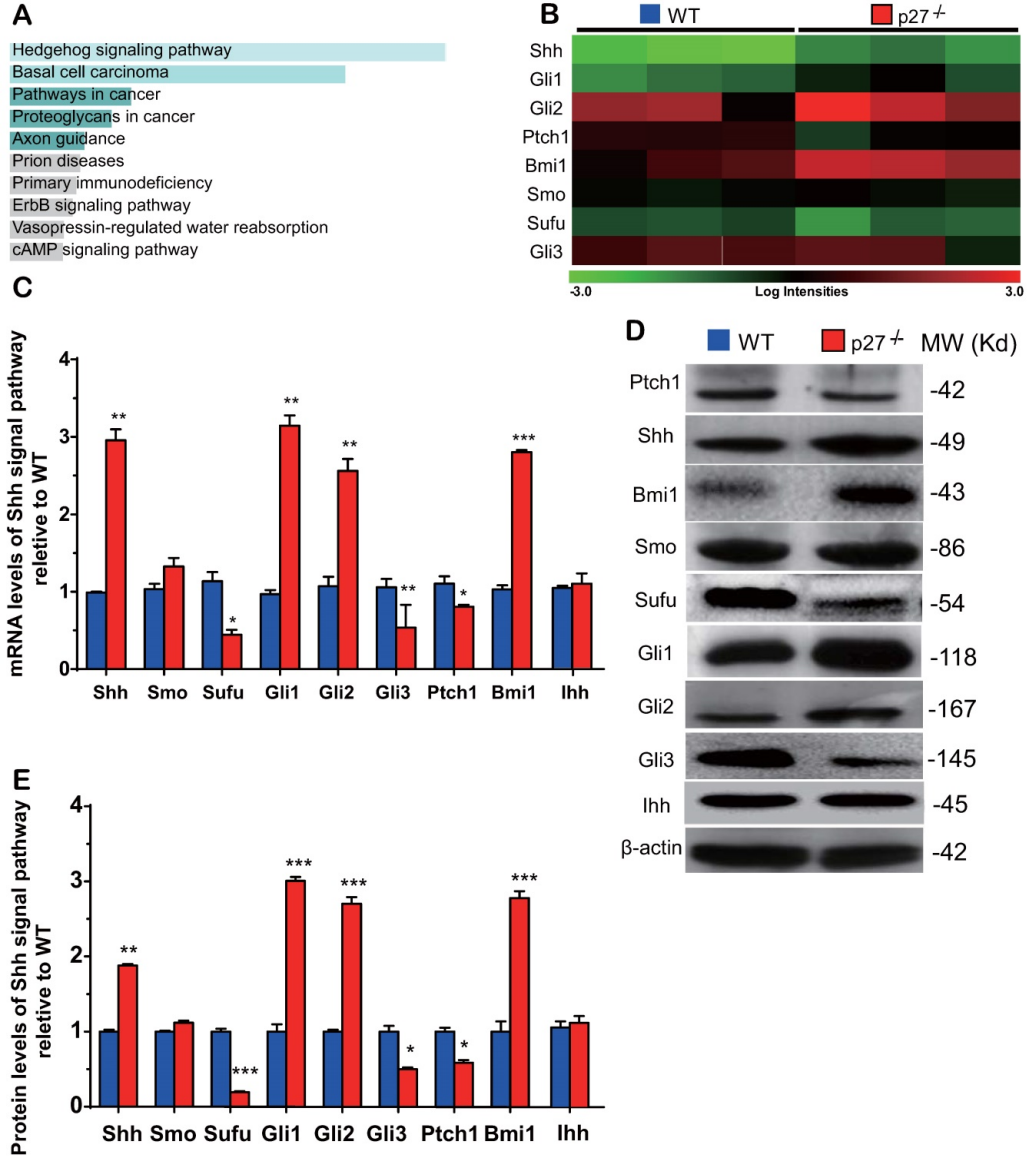
** Sonic hedgehog signaling pathway is activated in the bone tissue of p27 deficient mice. (A)** Kyoto Encyclopedia of Genes and Genomes (KEGG) pathways of differentially expressed mRNAs and **(B)** the heat map of Shh signaling genes differentially expressed in bone tissue of 3 WT and 3 p27^-/-^ mice. Confirmation of results of microarray using **(C)** real-time RT-PCR and **(D)** Western blots. **(E)** Relative protein expression levels of Shh signaling molecules. Values are mean ± s. e. m. of 3 determinations per group. *:* P*< 0.05, **:* P*< 0.01, ***:* P*< 0.001 compared with WT mice.

To identify the critical molecules which mediated osteogenesis enhanced by p27 deficiency, a microarray analysis was performed in wild-type and p27 deficient bone tissue using 44,170 mouse probes. The differential expression of 445 genes (DEGs) (>1.5-fold up- or downregulated genes, p < 0.05) was detected in the bone tissue of 4-week-old p27^-/-^ mice compared with their wild-type littermates (Supplemental Table 1). The Kyoto gene and genome pathway encyclopedia (KEGG) enrichment analysis was performed by using the Enrichr database 32, and results showed that 445 DEGs were significantly enriched in the hedgehog signaling pathway (Fig. 2A). The expression levels (log scale) of the molecules related to Shh signaling pathway were reordered and displayed in a heat map, with the spectrum ranging from green (low level) to red (high level), as presented in Fig. 2B. By real-time RT-PCR and Western blots we confirmed the alterations of Shh signaling related molecules detected by the microarray. At both mRNA and protein levels, the expression of Shh, Gli1 and 2 and their target gene Bmi1 was significantly up-regulated. The expression of smoothened (Smo) was up-regulated but insignificantly, and the expression of suppressor of fused (Sufu), Patched 1 (Ptch1) and Gli3 was significantly down-regulated in p27 deficient mice compared with wild-type littermates (Figs. 2C-E). We also examined the mRNA and protein expression levels of Indian hedgehog (Ihh) and found that they were not altered in p27 deficient mice compared with wild-type littermates (Figs. 2C-E). These results implied that p27 deficiency can activate the Shh signaling pathway in bone tissue.

### Shh signaling mediates p27 deficiency-induced osteogenesis *in vitro*

**Figure 3:**
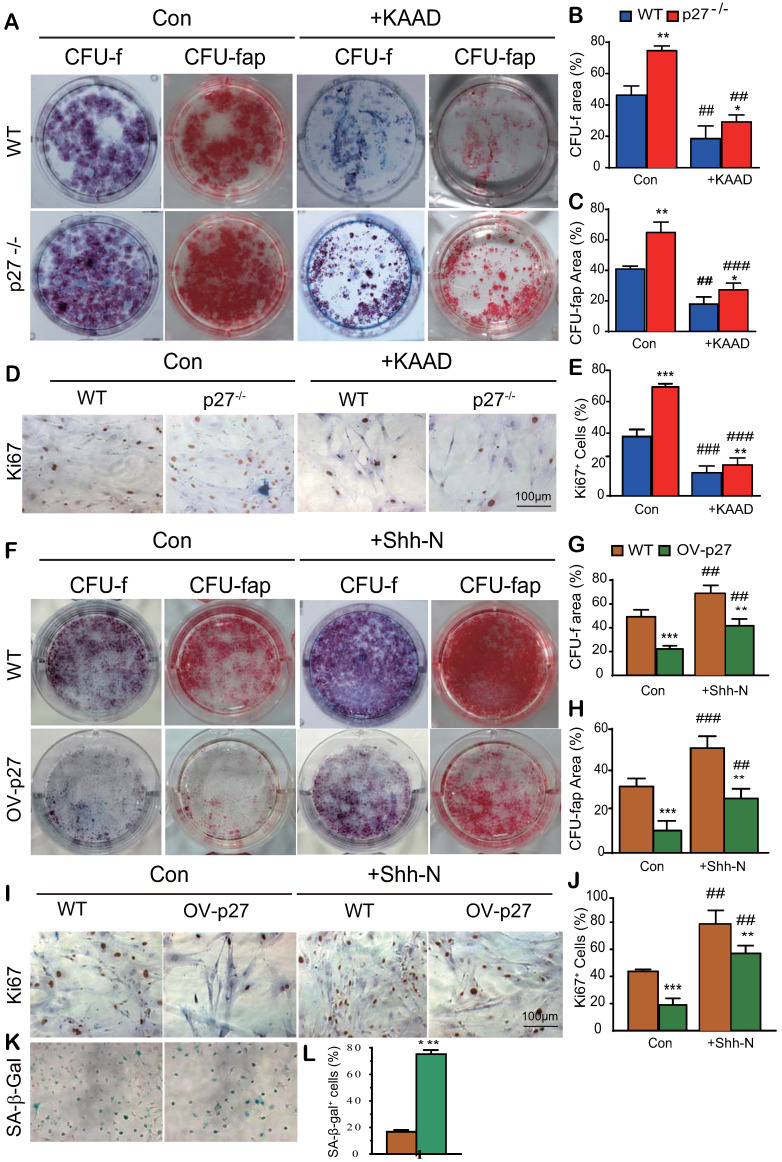
** Shh signaling mediates p27 deficiency-induced osteogenesis *in vitro*.** BM-MSCs derived from WT mice and p27^-/-^ mice were cultured in the absence or presence of 2.5 µM Shh antagonist KAAD-Cyclopamine (KAAD) for 10 days. Resulting cells were stained **(A)** with methyl blue or cytochemically for alkaline-phosphatase (ALP) to detect alterations of total CFU-f or ALP-positive CFU-f (CFU-fap), respectively. **(B)** CFU-f positive areas and **(C)** CFU-fap positive areas relative to culture dish areas. **(D)** Representative micrographs of immunocytochemical staining for Ki67 and **(E)** the percentage of Ki67 positive cells. BM-MSCs derived from wild-type mice were overexpressed with p27 and were cultured in the absence or presence of 200 ng/ml Shh-N for 10 days. Resulting cells were stained **(F)** for total CFU-f and CFU-fap. **(G)** CFU-f positive areas and **(H)** CFU-fap positive areas relative to culture dish areas. **(I)** Representative micrographs of immunocytochemical staining for Ki67 and **(J)** the percentage of Ki67 positive cells. **(K)** Representative micrographs of cytochemical staining for SA-β-gal and **(L)** the percentage of SA-β-gal positive cells. Values are mean ± s. e. m. of 3 determinations per group. *:* P*< 0.05, **:* P*< 0.01, ***:* P*< 0.001 compared with WT BM-MSCs. ##: *P*<0.01, ###:* P*<0.001 compared with genotype matched BM-MSCs treated with KAAD or Shh-N.

To explore whether Shh signaling mediated p27 deficiency-induced osteogenesis, BM-MSCs derived from wild-type or p27 deficient mice were cultured in the absence or presence of 2.5 μM Shh antagonist KAAD-Cyclopamine (KAAD) for 10 days. The cells were stained with methyl blue or cytochemically for alkaline-phosphatase (ALP) to detect alterations of total CFU-f or ALP-positive CFU-f (CFU-fap), respectively, and immunocytochemical staining for Ki67. Results showed that the percentages of both total CFU-f, CFU-fap areas and the percentage of Ki67 positive cells were significantly increased in p27 deficient BM-MSC cultures relative to wild-type BM-MSC cultures but were dramatically decreased in the p27 deficient BM-MSC cultures treated with KAAD (Figs. 3A-E). We then overexpressed p27 in BM-MSCs derived from wild-type mice and cultured them in the absence or presence of 200 ng/ml recombinant Sonic hedgehog N-Terminus Protein (Shh-N) for 10 days. We found that the percentages of both total CFU-f and CFU-fap areas and the percentage of Ki67 positive cells were significantly decreased in p27 overexpressed BM-MSC cultures compared to wild-type BM-MSC cultures and moderately increased in p27 overexpressed BM-MSC cultures treated with Shh-N (Figs. 3F-J). We also found that p27 overexpression significantly increased the percentage of senescence-associated β-galactosidase (SA-β-gal) positive BM-MSCs (Figs. 3K & L). These results indicate that Shh signaling mediated p27 deficiency-induced osteogenesis.

### Shh signaling mediates p27 deficiency-induced osteoblastic bone formation *in vivo*

**Figure 4:**
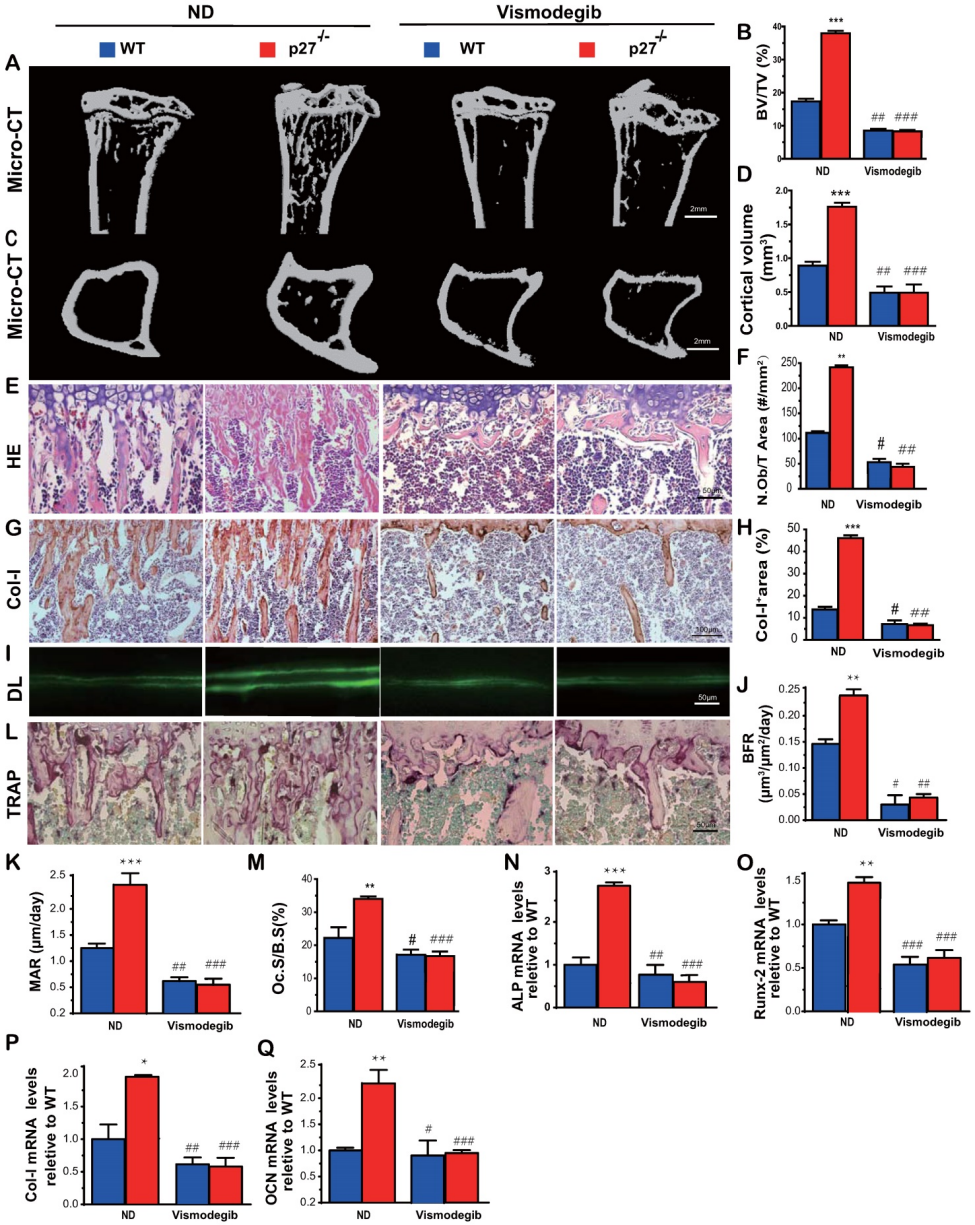
** Shh signaling mediates p27 deficiency-induced osteoblastic bone formation *in vivo*. (A, C)** Representative micro-CT scans of 3-dimensional longitudinal reconstructions of proximal ends of tibiae and midshaft diaphysis from 8-week-old WT and p27^-/-^ mice fed a normal diet (ND) or a diet with Shh antagonist Vismodegib for 2 weeks after weaning. **(B)** Trabecular bone volume relative to tissue volume (BV/TV, %). **(D)** Cortical bone volume (mm^3^). Representative micrographs of paraffin-embedded sections of tibias from 8-week-old WT and p27^-/-^ mice: **(E)** stained with H & E and **(F)** the number of osteoblasts per mm^2^ tissue area (N.Ob/T.Ar, #/mm^2^), **(G)** stained immunohistochemically for type I collagen (Col-I) and **(H)** Col-I-positive areas as a percent of the tissue area (%), **(I)** Double calcein labeling (DL), **(J)** bone formation rate (BFR, µm^3^/µm^2^/day) and **(K)** mineral apposition rate (MAR, µm/day), **(L)** histochemical staining for TRAP and **(M)** osteoclast surface/bone surface (Oc.S/BS, %). Real-time RT-PCR analyses of long bone extracts for the expression of **(N)** *ALP*, **(O)** *Runx2*, **(P)** *Col-I* and **(Q)** *OCN*. Messenger RNA expression assessed by RT-PCR was calculated as a ratio relative to the GAPDH mRNA level and expressed relative to WT control. Values are mean ± s. e. m. of 5 determinations per group. *: *P*<0.05; **: *P*<0.01 compared with WT mice. #: *P*<0.05; ##: *P*<0.01, ###:* P*<0.001 compared with genotype matched mice fed a normal diet.

To determine whether Shh signaling mediated p27 deficiency-induced osteoblastic bone formation *in vivo*, 8-week-old wild-type and p27^-/-^ mice were given the Shh antagonist vismodegib in their diet for 2 weeks, and their long bone phenotypes were then analyzed. The results showed that the trabecular and cortical bone volume (Figs. 4A-D), osteoblast number (Figs. 4E-F), type I collagen positive area (Figs.4G-H), bone formation rate and mineral apposition rate (Figs.4I-K), osteoclast surface (Figs.4L & M) and mRNA expression levels of *Runx2*, *ALP*, *type I collagen* and *osteocalcin* (Figs. 4N-Q) were all increased significantly in p27 deficient mice compared with their wild-type littermates on the normal diet, but these parameters were significantly decreased in vismodegib-treated wild-type and p27 deficient mice compared with mice of the same genotype on the normal diet. However, no significant differences in these parameters were detected between vismodegib-treated wild-type and p27 deficient mice. These results demonstrated that inhibition of Shh signaling can block p27 deficiency-induced osteoblastic bone formation *in vivo* and support the concept that p27 is a negative regulator of the Shh signaling pathway in bone tissue.

### Deletion of p27 releases Shh transcription suppression mediated via E2F4

**Figure 5:**
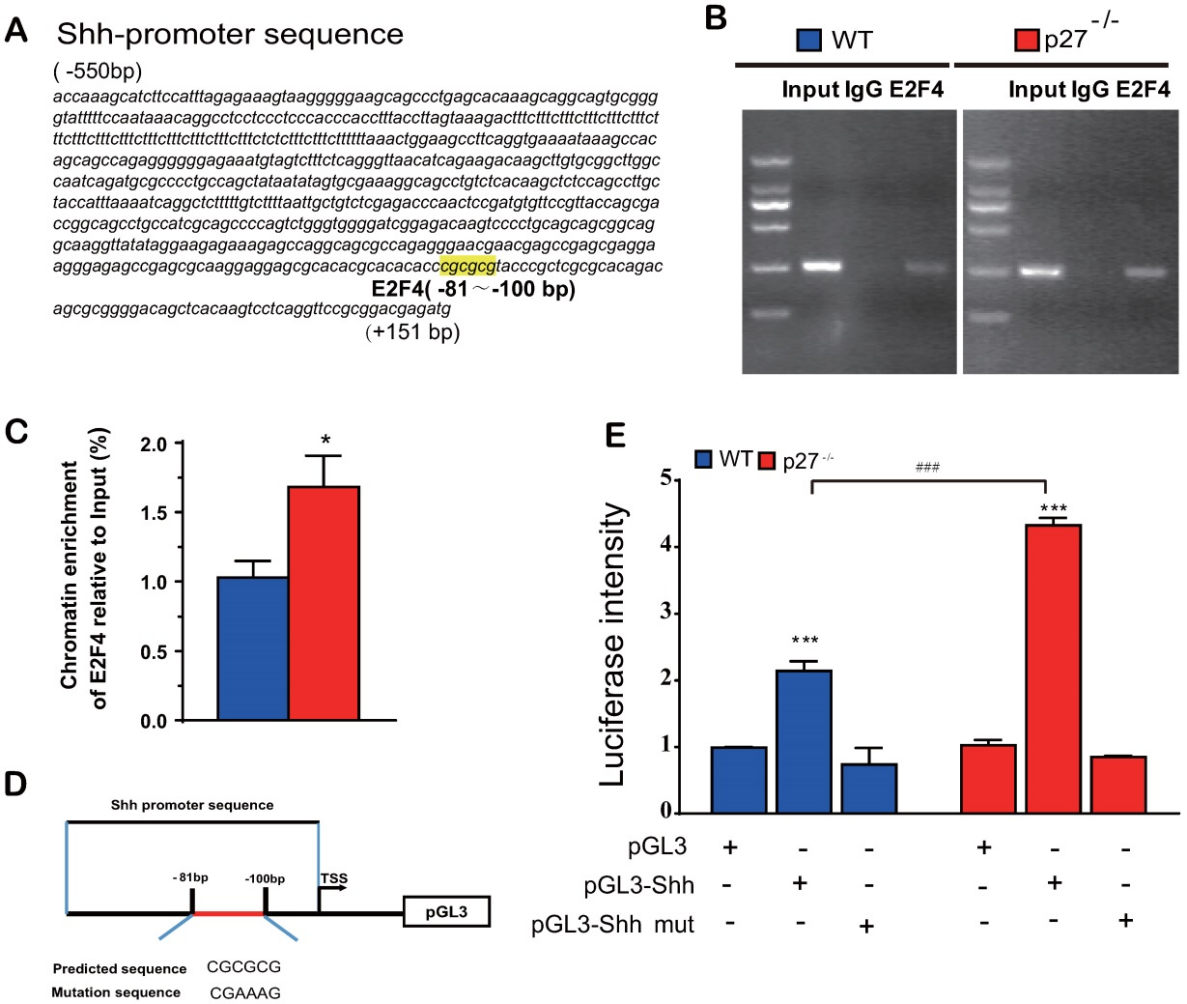
** Deletion of p27 releases Shh transcription suppression mediated via E2F4. (A)** A putative E2F4 binding site in the promoter region of the Shh gene highlighted in yellow color. **(B & C)** Analysis of E2F4 binding to Shh promoter using ChIP assays in wild-type and p27 deficient MEFs. *: *P*<0.05 compared with wild-type MEFs. **(D)** Schematic structural diagram of PGL3‐E2F4 promoter and mutant PGL3‐E2F4 Luc reporter plasmid. **(E)** Luciferase activity driven by the Shh promoter in wild-type and p27 deficient MEFs. Values are mean ± s. e. m. of 3 determinations per group. *: *P*<0.05; **: *P*<0.01 compared with the empty plasmid. #: *P*<0.05; ##: *P*<0.01, ###: *P*<0.001 compared with MEFs transfected with the same plasmid.

Previous studies suggested that p27 has a role as a transcriptional repressor in combination with p130 and E2F4. Immunoprecipitation experiments revealed that p27 co-immunoprecipitated with endogenous E2F4 and p130 2, however, it is unclear whether E2F4 mediates Shh transcription. To answer this question, we identified a putative E2F4 binding site in the promoter region of the Shh gene (retrieved from the NCBI mouse genome database) by computer‐assisted analysis (Fig. 5A). Using a ChIP approach, we confirmed that E2F4 had the ability to physically bind the Shh promoter in wild-type mouse embryonic fibroblasts (MEFs) and this binding was more enriched in p27 deficient MEFs (Figs. 5B & C). Luciferase assays showed that luciferase activities were increased significantly in wild-type MEFs transfected with PGL3‐E2F4 plasmid compared with the empty plasmid and were increased more dramatically in p27 deficient MEFs transfected with PGL3‐E2F4 plasmid. In contrast, luciferase activities were not increased in wild-type or p27 deficient MEFs transfected with PGL3‐E2F4 mutant plasmid compared with the empty plasmid (Figs. 5D & E). These results indicate that p27 acts as a transcriptional repressor in combination with E2F4 to suppress Shh transcription.

### Deletion of Bmi1 blocks p27 deficiency-induced osteoblastic bone formation *in vivo*

**Figure 6:**
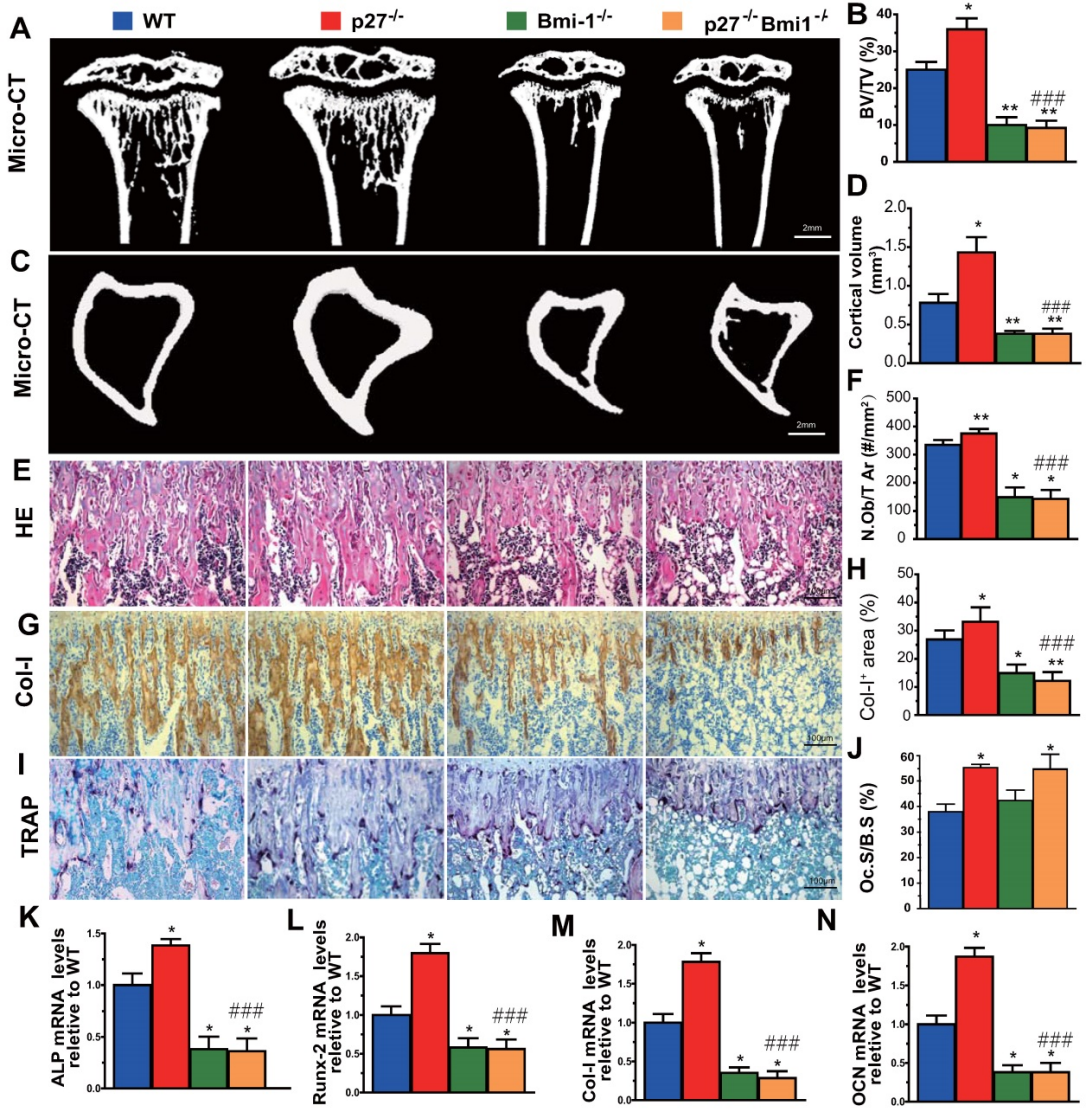
** Deletion of Bmi1 blocks p27 deficiency-induced osteoblastic bone formation *in vivo*. (A, C)** Representative micro-CT scans of 3-dimensional longitudinal reconstructions of proximal ends of tibiae and midshaft diaphysis from 4-week-old WT, p27^-/-^, Bmi1^-/-^ and p27^-/-^Bmi1^-/-^ mice. **(B)** Trabecular bone volume relative to tissue volume (BV/TV, %). **(D)** Cortical bone volume (mm^3^). Representative micrographs of paraffin-embedded sections of tibias from 4-week-old WT, p27^-/-^, Bmi1^-/-^ and p27^-/-^Bmi1^-/-^ mice: **(E)** staining with H&E and **(F)** the number of osteoblasts per mm^2^ tissue area (N.Ob/T.Ar, #/mm^2^), **(G)** immunohistochemical staining for type I collagen (Col-I) and** (H)** Col-I-positive areas as a percent of the tissue area (%), **(I)** histochemical staining for TRAP and **(J)** osteoclast surface/bone surface (Oc.S/BS, %). Real-time RT-PCR analyses of long bone extracts for the expression of **(K)** *ALP*, **(L)** *Runx2*, **(M)** *Col-I* and **(N)** *OCN*. Messenger RNA expression assessed by RT-PCR was calculated as a ratio relative to the GAPDH mRNA level and expressed relative to WT control. Values are mean ± s. e. m. of 5 determinations per group. *: *P*<0.05; **: *P*<0.01 compared with WT mice. ###:* P*<0.001 compared with p27^-/-^ mice.

To evaluate whether Bmi1 mediated p27 deficiency-induced osteoblastic bone formation *in vivo*, we generated p27^-/-^Bmi1^-/-^ double knockout mice and compared them with p27^-/-^, Bmi1^-/-^, and wild-type littermates at 4 weeks of age. We found that the trabecular and cortical bone volume (Figs. 6A-D), osteoblast number (Figs. 6E & F), type I collagen positive area (Figs. 6G & H), osteoclast surface (Figs. 6I & J) and mRNA expression levels of *Runx2*,* ALP*,* type I collagen* and* osteocalcin* (Figs.6K-N) were all increased significantly in p27 deficient mice, and were all, except for osteoclast surface decreased dramatically in Bmi1^-/-^ or p27^-/-^Bmi1^-/-^ mice compared with their wild-type littermates; in addition. these parameters except for osteoclast surface were significantly decreased in p27^-/-^Bmi1^-/-^ mice compared with p27^-/-^ mice (Fig. 6). The bone phenotype of p27^-/-^Bmi1^-/-^ mice closely resembled that of Bmi1^-/-^ mice (Fig. 6). These results suggest that Bmi1 deletion can block the activation of the Shh signaling pathway induced by p27 deficiency, and therefore can block p27 deficiency-induced osteoblastic bone formation *in vivo*.

## Discussion

Cyclin dependent kinase inhibitor p27 regulates cell proliferation, cell motility and apoptosis. The p27 expression levels in several tumor types have both prognostic and therapeutic implications 33. There are studies that have implied that p27 is also a critical factor in osteogenesis and osteoblastic bone formation 3-5, however, the mechanisms underlying its functions in bone metabolism is unclear. In this study, we first confirmed that deletion of p27 could enhance osteoblast formation and osteogenesis by comparing bone phenotypes of 4-week-old p27 deficient and wild-type littermates. To identify the critical molecules which mediated osteogenesis enhanced by p27 deficiency, we performed a microarray analysis in wild-type and p27 deficient bone tissue and the mRNA and protein levels of the molecules of interest were confirmed to be elevated or reduced by RT-PCR and Western blots. Our results demonstrated that deletion of p27 significantly upregulated the expression of Shh, Gli1 and 2 and their target gene Bmi1 and significantly downregulated the expression of Sufu, Ptch1 and Gli3 in bone tissue, but did not alter the expression level of Ihh. The critical function of hedgehog signaling in bone formation has been identified in the past two decades 34. Shh acts at early stages of development to regulate patterning and growth 14, and also enhances the proliferation and osteoblastic differentiation of BM-MSCs 16 and of periosteal-derived mesenchymal progenitor cells 17. Gli1-haploinsufficient mice exhibit reduced bone mass with impaired osteoblast differentiation 35. In addition, deletion of Gli2 impaired endochondral bone development and displayed osteopenia 36. Patch1 haploinsufficiency increases adult bone mass, and Ptch1-deficient osteoblast precursor cells differentiate into osteoblasts at an accelerated rate as a result of an enhanced response to Runx2 and by reducing the generation of the Gli3 repressor 37. Ligand-independent activation of hedgehog signaling in Sufu-deficient mice was similar to that observed in mice deficient in Ptch1 38. The deletion of Gli3 results in an increased ossification of calvarial bone, causing craniosynostosis 39. Taken together with the previous data demonstrating the important role of hedgehog signaling in bone formation, the results of the present study, suggest that deletion of p27 enhances osteoblast formation and osteogenesis by activating the Shh signaling pathway in bone tissue.

To further demonstrate that Shh signaling mediated p27 deficiency-induced osteoblast formation *in vitro*, BM-MSCs derived from wild-type or p27 deficient mice were treated with the Shh antagonist KAAD-cyclopamine, and in response to this antagonist, osteogenic capacity was reduced significantly in both wild-type and p27 deficient BM-MSCs, but especially in p27 deficient BM-MSCs. We also found that overexpression of p27 in BM-MSCs significantly reduced osteogenesis by BM-MSCs; by contrast, the Shh agonist Shh-N significantly enhanced osteogenesis of BM-MSCs, and this enhancement was reduced significantly by overexpression of p27. Vismodegib is a hedgehog pathway inhibitor indicated for the treatment of adults with metastatic basal cell carcinoma 40. When vismodegib was given in the diet to 8-week wild-type and p27 deficient mice for 2 weeks, the increased bone volume and osteoblastic bone formation parameters induced by p27 deficiency were completely blocked, *in vivo*. A previous study has demonstrated that inhibition of the hedgehog signaling pathway by cyclopamine treatment suppressed the expression of osteoblast-related genes and decreased bone mineralization, and that blocking hedgehog signaling through knockdown of Shh and Gli2 genes led to defective osteoblast differentiation; in contrast, promoting hedgehog signaling by knockdown of Ptch1 was beneficial to osteoblast differentiation 41. Results from our study provide evidence that deletion of p27 enhances osteoblast formation and osteogenesis by activating Shh signaling in bone tissue.

Previous studies suggest that p27 has a role as a transcriptional repressor in coordination with p130 and E2F4 2. Immunoprecipitation experiments revealed that p27 co-immunoprecipitated with endogenous E2F4, p130, mSIN3A and histone deacetylases, indicating that they form complexes *in vivo* 2. We detected a putative E2F4 binding site in the promoter region of the Shh gene, and using a ChIP approach, confirmed that E2F4 has the ability to physically bind the Shh promoter in wild-type MEFs and their binding was more enriched in p27 deficient MEFs. Luciferase assays demonstrated that the putative promoter region containing the predicted E2F4 binding sites of the Shh gene is sufficient to promote transcription of Shh, and Shh transcription was further enhanced by deletion of p27. Our results therefore indicate that p27 acts as a transcriptional repressor in coordination with p130 and E2F4 by suppressing Shh transcription via E2F4.

In previous studies we demonstrated that Bmi1 deficiency results in premature osteoporosis with reduced osteoblast formation and osteogenesis 10, whereas overexpression of Bmi1 in MSCs elicited antiosteoporosis effects by enhancing osteoblast formation and osteogenesis 11. Recently we found that 1,25(OH)_2_D_3_ upregulated Bmi1 expression at a transcriptional level via the vitamin D receptor; Bmi1 overexpression in MSCs corrected bone loss induced by 1,25(OH)_2_D deficiency and the bone anabolic action of exogenous 1,25(OH)_2_D_3_ administration was blocked by deletion of Bmi1; these studies implicated Bmi1 as a key downstream target of 1,25(OH)_2_D, which plays a crucial role in preventing bone loss induced by 1,25(OH)_2_D deficiency 11. Several lines of evidence have demonstrated that Bmi1 regulates self-renewal of stem cells through suppressing p16/Rb and p19/p53/p21 signaling pathways and DNA damage, decreasing oxidative stress and maintaining mitochondrial function 42-44. Furthermore, previous studies have also demonstrated that Bmi1 functioned as a downstream target of the hedgehog pathway 12, 13, 45. Treatment with Shh or overexpression of Gli1 or Gli2 resulted in a 6-fold increase in expression of Bmi1 in human mammary stem/progenitor cells and these effects were blocked by the hedgehog pathway specific inhibitor cyclopamine 46. Therefore, we considered whether Bmi1, as a critical downstream target of Shh, plays a key role in p27 deficiency induced osteoblast formation. Our results demonstrated that deletion of p27 also significantly upregulated Bmi1 expression at both mRNA and protein levels in bone tissue, whereas deletion of Bmi1 blocked p27 deficiency-induced osteoblastic bone formation *in vivo*. Results from this study indicate that p27 deficiency induced increased bone by activating Shh-Gli-Bmi1 signaling.

Although we demonstrated that a Sonic Hedgehog-Gli-Bmi1 signaling pathway is present in bone tissue and BM-MSCs, because we employed global knockouts of p27 and Bmi1 we cannot exclude the possibility that *in vivo* effects on bone metabolism partially result from their indirect actions. Bone-specific knockouts of p27 or Bmi1 could therefore be useful to identify direct versus indirect actions of p27 and Bmi1 in bone metabolism.

Overall, therefore, our results demonstrated that p27 deficiency induced bone anabolic action by activating Shh signaling and their target gene Bmi1, subsequently stimulating osteogenesis of BM-MSCs and osteoblastic bone formation; in contrast, deletion of Bmi1 blocked bone anabolic action induced by p27 deficiency and activation of Shh signaling. The results of this study indicate that the signaling pathway Shh-Gli-Bmi1 plays a critical role in p27 deficiency induced bone anabolic action, and suggest that Bmi1 may be an important therapeutic target for osteoporosis induced by activation of p27 signaling or inactivation of sonic hedgehog signaling.

## Supplementary Material

Supplementary table.

## Figures and Tables

**Table 1:** Primers used in this study for real time RT-PCR

Name	S/AS	Sequence	Tm	bp
p27	S	GGGCAGATACGAGTGGCAG	62	153
	AS	TGAGACCCAATTAAAGGCACC		
Bmi-1	S	ATCCCCACTTAATGTGTGTCCT	60	116
	AS	CTTGCTGGTCTCCAAGTAACG		
*ALP*	S	GGAGCACAGGAAGTTGGGAC	55	393
	AS	GCGTGCTTGAGCTGAAGCTA		
*RUNX2*	S	GTGACACCGTGTCAGCAAAG	55	356
	AS	GGAGCACAGGAAGTTGGGAC		
*OCN*	S	CAAGTCCCACACAGCAGCTT	55	370
	AS	AAAGCCGAGCTGCCAGAGTT		
*Col-I*	S	TCTCCACTCTTCTAGTTCCT	55	269
	AS	TTGGGTCATTTCCACATGC		
*GAPDH*	S	TGGATTTGGACGCATTGGTC	55	211
	AS	TTTGCACTGGTACGTGTTGAT		
*Shh*	S	AAAGCTGACCCCTTTAGCCTA	60	119
	AS	TGAGTTCCTTAAATCGTTCGGAG		
*Smo*	S	GTGCTGTCTACATGCCCAAGT	62	128
	AS	GCAACGCAGAAAGTCAGGC		
*Ptch1*	S	GCCTTCGCTGTGGGATTAAAG	61	118
	AS	CTTCTCCTATCTTCTGACGGGT		
*Gli1*	S	CCAAGCCAACTTTATGTCAGGG	61	130
	AS	AGCCCGCTTCTTTGTTAATTTGA		
*Gli2*	S	CAACGCCTACTCTCCCAGAC	61	155
	AS	GAGCCTTGATGTACTGTACCAC		
*Gli3*	S	CACAGCTCTACGGCGACTG	62	168
	AS	CTGCATAGTGATTGCGTTTCTTC		
*Sufu*	S	CGGACCCCTTGGACTATGTTA	60	182
	AS	CTTCAGACGAAACGTCAACTCA		
*Shh-promoter*	S	GGTACCGTGGCCACCTGTGATTATCC	60	280
	AS	GAGCTCTGAGGACTTGTGAGCTGTCC		

## Abbreviations

BM-MSCs: Bone marrow mesenchymal stem cells
p27: Cyclin-dependent kinase inhibitor 1B or p27^Kip1^
Bmi1: B-lymphoma Mo-MLV insertion region 1
p27^-/-^: p27 Knockout
Bmi1^-/-^: Bmi1 Knockout
ALP: Alkaline phosphatase
Col-I: Type I collagen
OCN: Osteocalcin
DEGs: Differential expressed genes
Shh: Sonic Hedgehog
Smo: smoothened
Sufu: suppressor of fused
Ptch1: Patched 1
Indian hedgehog: Ihh
Sonic hedgehog N-Terminus Protein: Shh-N
MEFs: Mouse embryonic fibroblasts
