# Supplementary material for: A Sonic Hedgehog-Gli-Bmi1 signaling pathway plays a critical role in p27 deficiency induced bone anabolism
Source: Int J Biol Sci. 2022 Jan 1;18(3):956–69. doi: 10.7150/ijbs.65954 (PMC8771843; doi:10.7150/ijbs.65954)
Supplement: Supplementary file 1 — Supplementary table. [file ijbsv18p0956s1.pdf]

Supplemental Table 1: Differential expression of genes (DEGs) between p27$^{-/-}$ and WT mice

Gene	p-Value	Fold change	p27$^{-/-}$ VS WT
Hif3a	0.006813774	1.9198179	up
Gfm2	0.048074663	2.056672	up
Gtf2ird1	0.011527861	1.7260659	up
Scml2	0.006767468	1.6966984	up
Phactr3	1.32E-02	1.5896102	up
Josd2	0.032291315	2.1579154	up
Tsen34	0.031220352	2.1341693	up
Ypel1	0.030278603	1.9805248	up
1700022K14Rik	0.012234127	2.1624827	up
Stx6	0.022591665	1.7559271	up
Ppp1r12c	0.00099608	1.5169258	up
Smc4	0.000341513	1.853598	up
Pole2	0.027008342	1.6195295	up
2700007P21Rik	1.91E-02	1.7063528	up
Dnajc1	0.011500709	1.671233	up
2810006K23Rik	0.033279218	1.7962182	up
Hbs1l	0.003407194	1.8127105	up
Gm10664	0.046649475	1.6082802	up
Eif2ak1	0.03434675	1.711598	up
4921534H16Rik	0.001433927	2.2525785	up
Scamp1	0.030321397	1.6704565	up
Slc39a8	0.032230157	1.7144119	up
4930581F22Rik	0.017603585	2.4115288	up
6030468B19Rik	0.039658662	1.5131792	up
Adk	0.030560704	1.5723747	up
BMI1	0.008023806	1.7473743	up
Spag9	0.04126587	1.6458555	up
Pde1c	0.042807814	1.5995237	up
Usp25	0.02842498	1.7701699	up
Dlx6os1	0.035242897	1.9911327	up
Cyb5r4	0.004205644	1.7764735	up
Rangap1	3.29E-02	1.9350888	up
Slc20a1	0.023770427	1.7847558	up
Ache	0.048093397	1.5083127	up
Kif18a	0.029764665	1.5028707	up
AU042671	0.004370066	1.545073	up
Ptpn5	0.013456148	1.7445385	up
A930006K02Rik	0.007842793	1.7534032	up
Trim2	0.007391815	1.6964	up
4930523C07Rik	0.004368249	1.6815472	up
Narg2	0.04345669	1.5971301	up

Cep152	0.011154918	1.5021973	up
Ank3	0.018799877	1.8827225	up
3830408C21Rik	3.76E-03	1.643649	up
Pnma2	0.004952461	1.8894969	up
Ubr4	0.04288284	1.5745407	up
Grik1	1.27E-02	1.5208843	up
Dpysl5	0.031067101	1.5641336	up
Prpf40a	0.01582676	1.6784904	up
Pbx1	0.015612504	1.5238736	up
Herc1	0.031743083	1.5670356	up
Fbxw2	0.023987606	1.6260377	up
Pax6	0.04976317	1.5026462	up
4930422G04Rik	0.010625852	1.562629	up
Gm10125	0.023554841	1.6054553	up
Phf20l1	0.022313602	1.6682572	up
Gad1	0.026926296	2.0303092	up
Cltc	0.017984144	1.7226907	up
Scfd1	0.032045715	1.7493384	up
Nfxl1	0.029962901	1.5298336	up
Pbx3	0.036291458	2.72911	up
Tubgcp6	0.011564284	1.5050344	up
Myom1	0.024919355	1.5505034	up
Ano1	0.022994816	1.6297314	up
9630013D21Rik	0.030266207	1.5367644	up
E030019B13Rik	0.036404736	1.5858864	up
2900092D14Rik	5.00E-02	1.5208743	up
Ntng1	0.001869703	1.6714325	up
Whsc1	0.018748114	1.6324166	up
Acta2	0.002835201	2.283572	up
D430019H16Rik	0.011399334	1.7220901	up
Sec16a	0.002065038	1.5225384	up
Sgms1	0.018265389	1.8984824	up
1500004A13Rik	0.022576937	1.9140574	up
Kidins220	0.04017319	1.6130106	up
Cdc2l5	0.015564305	1.6571245	up
Fanci	0.026586007	1.6740841	up
Ralgapa2	0.002649085	1.6978652	up
Sh3glb1	0.03650072	1.5150739	up
Mtap2	0.001746766	1.8181847	up
Kctd9	0.039712653	1.6902101	up
Nfib	0.026297161	1.7240595	up
Ttc3	0.007590866	1.7065998	up
Camkk2	0.02279199	1.9318615	up
Phf16	0.00934503	1.5218512	up

Zfp2	0.042938553	2.0544083	up
Phf13	0.043404307	1.7998159	up
Adamtsl1	0.014694111	1.5254096	up
Cpt1c	0.019335708	1.5719705	up
Fbxo25	7.45E-03	1.5688069	up
Agrn	4.99E-02	1.988508	up
St18	0.047575947	1.6935782	up
Dennd2c	0.04983152	1.5309565	up
Pard3	0.009653779	1.6117215	up
G2e3	0.025907952	2.089872	up
Gtf2a2	0.038342632	1.7607752	up
Pip4k2c	0.028222697	1.7031034	up
Mpp2	0.004660428	1.5812099	up
Csf3r	0.006535446	1.5378277	up
Fut10	0.003743915	1.6481959	up
Traf3	0.034423735	1.5169008	up
Adc	2.54E-02	1.7211494	up
Eng	0.019119276	1.5347933	up
Atp8a1	0.037308857	1.5766284	up
Vps37d	0.031638585	1.7996309	up
Vps13c	0.009043626	1.7164564	up
Tatdn1	0.030202426	1.5101898	up
Tmem41b	0.020038074	1.5459619	up
Clcn3	6.74E-03	1.7466346	up
Dtl	0.034638982	1.931626	up
Ralgps2	0.004006786	2.385793	up
Fam78a	0.015391202	1.588701	up
Supt4h1	0.04283668	1.6624799	up
Pggt1b	2.20E-02	1.6319742	up
Atp6v0a1	0.029006097	1.5117065	up
Rptor	0.006292053	1.8530173	up
Krba1	0.04361059	1.5995083	up
Hs2st1	0.048081025	1.6417482	up
Phactr4	0.016076382	1.5597383	up
Klhl2	0.033217855	1.5390202	up
Cd200r3	0.013836836	1.9967722	up
Mier1	3.63E-02	1.5799477	up
Mpst	0.037419457	1.5703481	up
Cep290	0.0351727	1.5978186	up
Fzr1	0.010625714	1.565763	up
Epb4.1l4a	0.029593563	1.59694	up
Ppp1r14a	0.02241092	1.5889751	up
Hebp1	0.031018713	1.5806602	up
Banp	0.048462816	1.5426905	up

Sfrp2	0.012606891	1.633272	up
Acsl6	0.000778796	1.5931095	up
L2hgdh	0.04302605	1.708312	up
Car15	0.002280915	1.554754	up
Sostdc1	0.018604796	1.8455147	up
Slc6a9	0.039542343	1.5492744	up
Slc16a14	0.013769816	1.5961004	up
Dcaf8	2.01E-02	1.9857337	up
Nup133	0.013619601	1.5246464	up
Depdc1a	3.40E-02	1.6380559	up
1700054N08Rik	0.004812595	1.5713855	up
Optn	0.016817195	1.5234989	up
Lrrc8c	0.003198938	1.6103817	up
Bola1	0.00455713	1.6746883	up
Setd4	0.04259452	1.5727671	up
Nefl	0.002748787	2.0225625	up
Nmral1	0.010667702	1.5595568	up
Pcdhb20	2.08E-02	1.7989018	up
Fgfr4	0.03807412	3.6547275	up
Spc25	0.004565729	1.667323	up
Fam174b	0.009689888	1.7199234	up
Fnbp1l	0.021523325	1.5603911	up
Evc2	0.025644708	1.7700487	up
Mrpl20	0.010350404	1.5302736	up
Kifap3	0.024536425	1.8255872	up
Kdm5c	0.006100858	1.5829114	up
1700109H08Rik	2.52E-02	1.5279427	up
Zcchc12	0.035802517	1.5136443	up
Actl6b	0.042984024	1.5929363	up
Asb17	0.002262987	2.2164958	up
Gm550	6.72E-04	1.6212245	up
Vrk1	0.015476056	1.5710655	up
Sult4a1	0.008902832	1.6617827	up
Kcnb1	0.022830954	1.5121417	up
Cd226	0.002536955	1.56667	up
Phf2	8.71E-03	1.5304855	up
Adck5	0.045307472	1.6140233	up
Heph	2.16E-02	1.5720904	up
Gria1	0.04063761	1.9808527	up
Tbc1d16	0.047542855	1.9026843	up
Shb	0.041730467	1.8957767	up
Mid1	0.004482281	2.0982378	up
Chl1	1.71E-02	1.5389154	up
Trio	4.61E-02	1.7885275	up

5133400G04Rik	0.02927075	1.6714579	up
Zbtb12	0.002799203	1.5916327	up
Shh	0.003702286	2.046396	up
Zmym1	0.02498732	1.6778214	up
Kif24	0.004092569	1.8327355	up
Mars	0.04523676	1.5666969	up
Eef1d	0.00167156	1.8063601	up
Nrsn2	0.007324749	1.535694	up
D8Ertd82e	0.039315633	1.7385659	up
Mgat5b	0.017002417	1.9778466	up
Cxcr6	0.030320175	1.5409533	up
Zfp526	0.025356343	1.7271359	up
Msc	5.39E-03	1.6763337	up
Syn3	2.92E-02	1.9224021	up
Zfp398	0.03429047	1.646447	up
Traf3ip1	0.003910691	1.5297582	up
9630041N07Rik	3.14E-02	1.8766905	up
Prelid2	4.52E-02	1.5318474	up
Igsf1	0.032246884	1.5502374	up
Pin4	0.002618992	1.7725809	up
Trpc4	0.02514826	2.1032279	up
Rab37	0.038334012	1.7823943	up
Rwdd3	3.71E-02	1.5742462	up
Gli1	0.0333512	1.7208974	up
Rnf43	0.023926888	1.9541423	up
Fbxl16	0.037575155	1.8992124	up
Rai1	1.89E-02	1.7145332	up
Olfr356	3.43E-02	1.5809271	up
Mon1b	0.023545753	1.9901531	up
Trappc10	0.009988194	1.5996057	up
Lrp8	0.016690513	1.5094159	up
Lrfn1	0.031439297	1.8431647	up
Cenpf	0.00501913	1.5344753	up
Ska1	0.03927345	1.6913077	up
Slc24a2	0.040012818	1.7051401	up
A630033E08Rik	1.51E-03	1.5128121	up
Comt1	2.01E-02	1.5077305	up
Caprin1	1.21E-02	1.7235683	up
Cdkn1c	0.043793436	1.7526987	up
Fignl2	0.011035137	1.6060905	up
1700008J07Rik	0.022866057	1.5581483	up
E130102H24Rik	4.37E-02	1.5013618	up
Gm3323	3.87E-02	1.5674521	up
Gm3363	0.017088987	1.5079702	up

D430020J02Rik	0.027515756	1.7787156	up
Gm3793	0.027681686	1.580609	up
Gm3902	0.04267927	1.7090073	up
Gm4005	4.69E-02	1.8161126	up
Gm4589	1.34E-02	1.6096437	up
Zbtb11	0.039642666	1.6794349	up
Six3os1	0.017700527	2.6347125	up
Rnf165	0.010548617	1.5488459	up
Gm6661	3.55E-02	1.6210086	up
Cdkn3	0.02276836	1.9105551	up
Wnk3	1.69E-02	1.7973477	up
AI854517	2.10E-02	2.0270143	up
Gm8122	0.039627153	1.727499	up
Gm5843	0.042833216	1.6195155	up
Hoxa3	0.009476123	1.9291255	up
GLI2	0.030570898	1.9562594	up
SMO	0.019864157	1.1717683	up
Cav2	0.011254692	1.5810964	down
Sec16b	0.007784648	1.667091	down
Sirpb1	5.35E-03	1.6332817	down
Camk2b	0.012783773	1.8584719	down
Arhgap6	0.027896946	1.7030832	down
Clip1	2.52E-02	1.6160315	down
Rgs14	0.012158901	2.5456572	down
1700123M08Rik	0.046681587	1.797492	down
1110020A21Rik	0.017764922	1.8466129	down
Fam35a	0.034668148	1.5496222	down
4921525O09Rik	0.007947727	2.0384862	down
Mllt4	0.001347493	1.5397071	down
Orly	0.00324209	1.8372831	down
5031434O11Rik	0.015025012	1.7791466	down
Col27a1	7.19E-03	1.6936744	down
Pde7b	0.037070427	1.5184733	down
Ddx3y	0.030008448	1.7068537	down
Lpp	0.044938993	1.5216612	down
Zmym6	0.0248313	2.1685143	down
D930016D06Rik	3.57E-02	1.5317036	down
Ifi203	0.023343824	1.6095761	down
Atg2a	0.007521004	3.1187139	down
Ankrd35	0.04053417	1.6417829	down
Tm6sf1	0.006634045	1.6672502	down
Rps6kc1	0.030508827	1.8060861	down
Sgca	0.019845283	2.3395438	down
Pias4	0.008700271	1.7062107	down

Golim4	0.013698566	1.605226	down
Elk1	0.021999974	1.6259598	down
Fam175b	0.02087558	1.5071656	down
D930048N14Rik	0.002669771	1.959929	down
Nr4a2	1.07E-02	1.5717517	down
Smpd2	0.005064898	1.5949556	down
Kcnk13	0.009893609	1.6829817	down
Rhobtb2	0.009417764	1.571666	down
Slc7a6os	0.00636117	1.7180996	down
Vps33b	0.017532343	1.5756564	down
Pdlim2	0.008752701	2.1171267	down
Ciita	0.009178646	1.7869687	down
Gm1008	0.045903392	1.7266843	down
Nat10	0.015212216	1.5176494	down
Acacb	0.015930234	1.6100901	down
Mynn	0.003547225	1.6524581	down
Pstpip2	0.037859164	1.7150189	down
B230312C02Rik	0.035776205	1.5453473	down
Rgnef	0.008927009	1.7358438	down
Crebl2	0.006955992	1.7365364	down
1700052N19Rik	0.019165669	1.804599	down
Smtnl1	0.034256965	1.5026025	down
Scamp2	0.009161644	1.7450056	down
Cpxm1	0.038254738	1.5010518	down
Rabep2	7.96E-04	1.5071177	down
Ttc30a1	0.04720361	1.6031778	down
3200002M19Rik	0.023400856	1.7047192	down
Igh	0.03694332	2.9103131	down
Creb3l3	0.03920853	1.5843288	down
Rbbp9	0.001521936	1.5644697	down
Qprt	0.04137094	1.7106218	down
Cdkn1b	0.047972076	2.3650393	down
Frzb	0.011413178	2.088803	down
Fcna	0.018749839	1.5370244	down
Atf3	0.036747675	2.4913125	down
Ttll3	0.000127435	1.6840912	down
D4Bwg0951e	0.020575535	1.5559317	down
Tfpi2	0.004865132	1.9955555	down
Perp	0.008289864	1.529645	down
Aqp7	0.03818031	1.5668812	down
P2rx3	0.032792516	1.620707	down
Xlr4b	4.33E-02	1.5461386	down
Jak3	0.033414863	1.8806745	down
P2ry6	3.68E-02	1.7051193	down

Casp12	0.018841758	2.0141523	down
Dbndd2	0.011783659	1.5335748	down
Bglap-rs1	1.15E-02	1.5273781	down
Slco4a1	0.017364454	1.7352546	down
Paox	0.018337503	1.5011247	down
Aspn	0.034345347	1.6275184	down
Lynx1	0.01713467	1.8086661	down
Acot11	0.029503943	1.7715969	down
Rsph1	1.10E-02	1.549216	down
Actg2	0.01317732	1.6675996	down
4933411K16Rik	0.029858723	1.5800297	down
Cyp2j6	0.002688697	1.5162892	down
Tor3a	0.005020795	2.1834345	down
Cd96	0.03207914	1.9108084	down
Pld4	0.020958748	1.6556984	down
Fmod	2.49E-02	1.7696937	down
Trpc6	0.012838276	1.8765584	down
H2-K1	5.02E-03	1.6752896	down
Myo16	3.56E-02	1.5135589	down
Zfp449	0.036487278	1.5902839	down
1110051M20Rik	0.003462219	1.559447	down
Csgalnact1	0.047053143	1.7436081	down
3110057O12Rik	0.010632735	1.5604407	down
Tmem177	0.04939468	1.5098695	down
Ly9	0.008945195	1.9302589	down
Card11	0.036025047	1.521121	down
Refbp2	2.80E-02	1.5063359	down
Tsnaxip1	2.23E-02	1.55527	down
Serpinb1c	0.010608555	1.9827956	down
1700029J07Rik	0.041937236	1.770397	down
Zfp641	0.023553038	1.556817	down
Slc2a12	0.04868283	1.8123457	down
Mepe	2.46E-02	1.584907	down
Vpreb1	0.000317106	2.2577403	down
2310046A06Rik	0.003520815	1.7502048	down
Mnda	3.75E-02	1.6727124	down
Cox18	0.04216266	1.5927383	down
Bag3	0.01391275	1.6499329	down
SUFU	0.03364187	1.9737358	down
Rnase12	3.32E-02	1.6488485	down
Vpreb2	0.048572533	1.9762888	down
Rspo2	0.014198713	1.8794149	down
Trim63	0.016715558	1.6659915	down
Gm6316	1.21E-02	1.641121	down

Aldoart1	0.03568287	4.0655413	down
Aldoart2	0.034349777	2.2996223	down
Ly6a	2.37E-02	1.7925441	down
Amy2b	1.76E-02	1.8923837	down
LOC547349	4.13E-02	3.454701	down
Zfp831	0.03961507	1.7062659	down
Cyp2c69	3.00E-02	2.2772725	down
Cbfa2t3	0.037971817	1.6317364	down
Abhd14a	0.028818158	1.6859397	down
Amy2a5	4.13E-03	2.0677986	down
Klra23	4.68E-02	2.1195457	down
Gm7027	0.04483205	1.685265	down
Hoxa11as	0.023755506	1.7007066	down
Aqp4	0.020595202	1.5613228	down
Gm12617	3.66E-02	4.2030272	down
Gm9339	0.010502324	1.8527687	down
Gm5784	0.024868064	1.6172887	down
Gm2025	1.82E-02	2.6562324	down
Gm9776	3.78E-02	1.8087558	down
Gm2212	4.48E-02	1.5303667	down
Gm2289	1.20E-02	1.8949467	down
Gm9808	0.016375463	3.093326	down
B130034C11Rik	1.11E-03	2.0175624	down
Gm9457	3.30E-02	1.5465527	down
Gm2414	0.031716023	1.7997339	down
Gm2556	0.01261517	1.8734963	down
Gm5222	1.69E-02	1.7702839	down
Gm10270	3.01E-02	2.1994097	down
Gm2640	0.020552106	2.5460064	down
Gm2581	4.05E-02	2.198683	down
Gm2724	8.16E-03	1.8851714	down
Gm2758	0.026631974	1.6839665	down
Gm2648	0.032429848	1.5828382	down
Gm2917	3.51E-02	1.8304363	down
Gm2797	0.041068833	1.6884123	down
Gm5698	0.021672675	2.657222	down
C920006O11Rik	6.19E-05	3.2880456	down
Gm3301	0.011949386	2.7820358	down
Gm3219	0.00123454	1.6451725	down
Gm3518	1.97E-03	1.8004127	down
Gm11938	2.93E-02	2.3881085	down
Gm3804	1.64E-02	1.5576179	down
Gm3845	0.034088433	2.1645699	down
Gm3884	3.86E-02	1.5455112	down

Gm6506	0.033121668	1.6462843	down
Gm11589	0.01096218	1.9746552	down
Gm10237	3.13E-02	2.4008148	down
Gm5881	0.030925676	1.9964178	down
Gm12770	0.03534486	1.8351032	down
Smt3h2-ps2	0.03593512	1.5447472	down
Gm4529	2.38E-02	3.1086211	down
Gm9627	0.024913097	1.6314678	down
Gm4588	0.005130137	2.0247645	down
Gm4603	0.016977923	2.1975808	down
Gm10077	0.028962163	2.1005323	down
Gm4703	0.0365702	1.541587	down
Gm9462	0.03332867	1.7291676	down
Gm4986	4.32E-03	1.7182899	down
Gm4963	0.016531017	2.3504543	down
Gm4888	0.035646074	1.952753	down
Gm6579	3.36E-02	1.6631883	down
Lpar5	0.023394685	1.8726568	down
PTCH1	0.008023806	1.7473743	down
Gm5258	0.014624729	2.9415436	down
Gm5265	0.026374469	1.6885926	down
Gm5278	0.023607558	2.0393746	down
Gm5449	4.90E-02	1.9021354	down
Rpl26l1	2.49E-03	1.6957425	down
GLI3	0.048890356	1.5896938	down
Gm13880	5.04E-03	2.0530453	down
Gm5670	0.045809694	2.8745923	down
Gm5699	0.024250697	2.1216083	down
Gm13843	0.019613106	2.806521	down
Glud2	0.02562094	1.9138448	down
Gm5927	0.042043135	1.8997698	down
Gm6141	0.011896855	1.963482	down
Gm6109	4.85E-02	2.313615	down
Gm6186	0.026456695	1.9758996	down
Gm6259	1.49E-02	2.4817355	down
Gm6272	0.0300803	4.7019205	down
Gm6378	0.03572597	2.1870966	down
Gm6665	0.021585941	1.5066156	down
Gm6790	2.36E-03	2.5876424	down
Gm6816	4.79E-03	2.1657379	down
Gm6932	0.04554543	1.8720732	down
Gm7288	0.029340666	1.7335596	down
Gm7574	4.33E-02	1.9394716	down
Gm7303	0.024536874	1.9788014	down

Gm4978	0.018426623	2.8667874	down
Gm8024	1.90E-03	1.6958255	down
Gm8055	0.022624057	1.6781082	down
Gm8062	0.030168625	1.5916201	down
Gm8882	2.16E-02	2.2502072	down
Gm8951	0.017455403	1.7473725	down
Gm9034	3.24E-02	2.5723088	down
Gm5507	0.026388202	1.8448468	down
